# The history and value of face masks

**DOI:** 10.1186/s40001-020-00423-4

**Published:** 2020-06-23

**Authors:** Christiane Matuschek, Friedrich Moll, Heiner Fangerau, Johannes C. Fischer, Kurt Zänker, Martijn van Griensven, Marion Schneider, Detlef Kindgen-Milles, Wolfram Trudo Knoefel, Artur Lichtenberg, Bálint Tamaskovics, Freddy Joel Djiepmo-Njanang, Wilfried Budach, Stefanie Corradini, Dieter Häussinger, Torsten Feldt, Björn Jensen, Rainer Pelka, Klaus Orth, Matthias Peiper, Olaf Grebe, Kitti Maas, Edwin Bölke, Jan Haussmann

**Affiliations:** 1 Department of Radiation Oncology, Heinrich-Heine-University, Duesseldorf, Germany; 2 Department of the History, Philosophy and Ethics of Medicine, Heinrich-Heine-University, Duesseldorf, Germany; 3 Institute for Transplant Diagnostics and Cell Therapeutics, Heinrich-Heine-University, Duesseldorf, Germany; 4 University Witten/Herdecke, Center for Biomedical Education and Research (ZBAF), Witten, Germany; 5 Department cBITE, Maastricht University, MERLN Institute for Technology-Inspired Regenerative Medicine, Maastricht, The Netherlands; 6 Department of Experimental Anesthesiology, University of Ulm, Ulm, Germany; 7 Department of Anesthesiology and Intensive Care Medicine, Heinrich-Heine-University, Duesseldorf, Germany; 8 Department for General Visceral and Pediatric Surgery, Heinrich-Heine-University, Duesseldorf, Germany; 9 Department for Cardiac Surgery, Heinrich-Heine-University, Duesseldorf, Germany; 10 Department of Radiation Oncology, University Hospital LMU Munich, Munich, Germany; 11 Department of Gastroenterology, Hepatology and Infectiology, Heinrich-Heine-University, Duesseldorf, Germany; 12 Institute for Applied Statistics, Munich, Germany; 13 University of Hannover, Hannover, Germany; 14 Heinrich-Heine-University, Duesseldorf, Germany; 15 Department of Cardiology, Rhythmology and Intensive Care Medicine, Evangelical Hospital, Duesseldorf, Germany

**Keywords:** Surgical mask, Pandemic crisis, Wound infection, Behavior, Viral transmission, Bacterial transmission, History of medicine

## Abstract

In the human population, social contacts are a key for transmission of bacteria and viruses. The use of face masks seems to be critical to prevent the transmission of SARS-CoV-2 for the period, in which therapeutic interventions are lacking. In this review, we describe the history of masks from the middle age to modern times.

## Background

In last few months, many communications were brought to the public that face masks are ineffective during a pandemic crisis. Since April 27, 2020 face masks have become mandatory for shopping and in public transportation in Germany. In the Netherlands, it became mandatory only for public transportation, from June 1, 2020 onwards. However, in Asian countries people have been wearing masks in public for ages. Although New York and Hong Kong are both metropolitan areas, the corona virus pandemia was devastating in the US and not in Hongkong. This fact alone implies a necessary, and a more distinguished view of the normative application of facemasks. In two manuscripts, we are now describing the use of masks during this viral pandemic. This first review describes the history of facemasks. The second will concentrate on benefits and risks by wearing facemasks in modern times.

## Review

### “The surgical face mask has become a symbol of our times” [1]

On March 17, 2020, this headline appeared in the New York Times on an article regarding the role of face masks in times of the COVID-19 outbreak. This is the most recent expression of the use of face masks. However, face masks have been used since the middle ages.

### Middle ages to renaissance

**Fig. 1:**
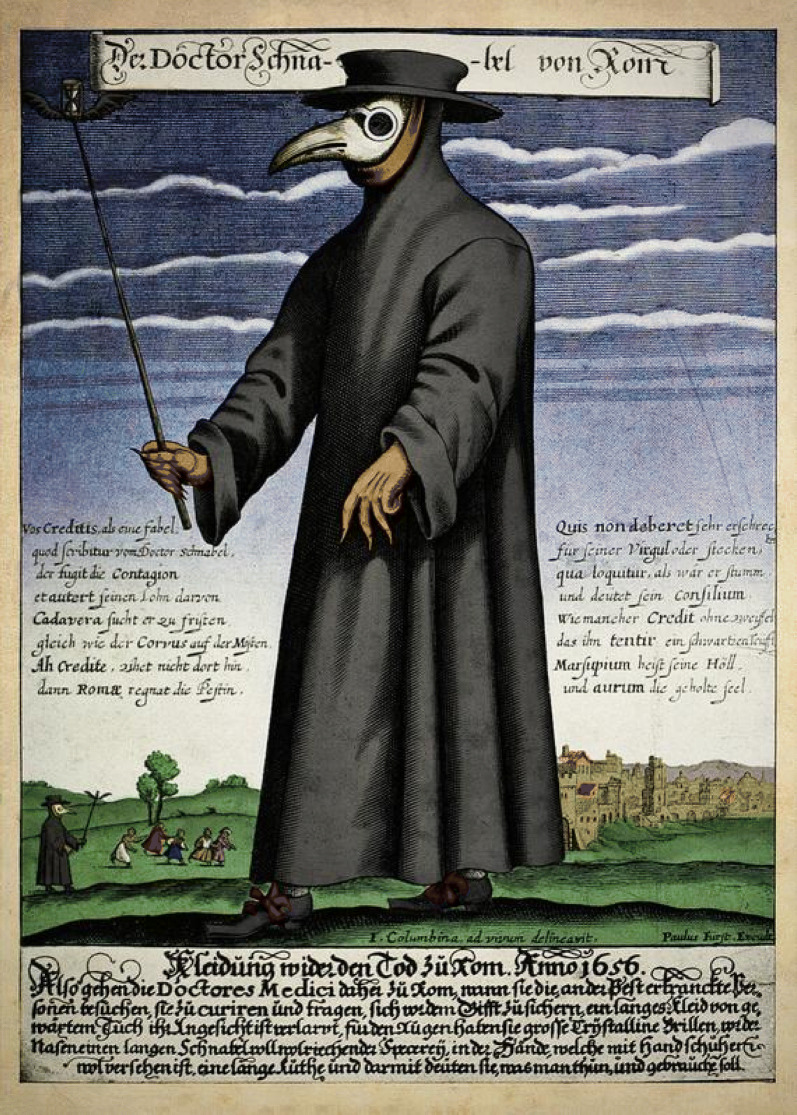
Colored version of a copper engraving of Doctor Schnabel (i.e., Dr. Beak), a plague doctor in seventeenth-century Rome, circa 1656 by Paul Fürst (1608–1666) of Nuremberg made for a broadsheet, German derivate of a sheet of Sebastiano Zecchini, 1656 (source Wikipedia https://de.wikipedia.org/wiki/Pestdoktor#/media/Datei:Paul_Fürst,_Der_Doctor_Schnabel_von_Rom_(coloured_version).png)

There are pictures of medical professionals from the early modern age treating patients suffering from the bubonic plague wearing beak-like masks. These masks were supposedly filled with herbs such as clove or cinnamon as well as liquids and led to the term ‘beak-doctors’ [2] (Fig. 1). The doctors were dressed in black cloaks and dark hats and were considered the symbol of the deathly epidemic of the Middle Ages. Their masks were meant to protect from the ‘blight’, the miasma, which was considered the cause of the plague back then. It was proclaimed that spoiled air from the East had caused the epidemic. Nevertheless, there is no proof that these ‘plague-doctors with beak-like masks’ really existed. There are two masks displayed in German museums that are suspected to be forgeries from a younger date. That indicates that the beak-doctors were in retrospect awarded a meaning they apparently did not have in reality [3].

### 1800–1900

Heroic stories of the introduction of antisepsis by Joseph Lister (1827–1912) and the corresponding preliminary works by Louis Pasteur (1822–1895) or Ignaz Semmelweis (1818–1865) [4] have inspired movie productions for decades and had an impact on our culture of remembrance. In contrast, the bacteriologic era that influenced the development of surgery has only recently been analyzed for the German area by Schlich et al. [5]. Ever since the works of Lister and Pasteur, the surgical ward and its developing special disciplines were confronted with a trend-setting discourse about wound infections and their prohibition and containment. This began in 1870, as the ‘hospital gangrene’ was limiting the outcomes of operations, especially those concerning abdominal procedures and those involving bones.

The introduction of mouth and nose coverage (mouth protection, face veils, face masks, mouth bandages) can be followed back to the turn-of-the-20^th^-century.

In 1897, the hygienist Carl Friedrich Flügge (1847–1923) working in Breslau at this time published his works on the development of droplet infections [6–8] as part of his research on the genesis of tuberculosis [7]. At that time, the respiratory system as a transmitter of germs came into focus of research and already mandated instructions to keep distance [7, 9]. In the same year, 1897, a cooperation work between Flügge and Theodor Billroth’s (1829–1894) disciple Johannes von Mikulicz (1850–1905), who also worked in Breslau since 1890, was published. Their publication dealt with performing operations wearing a ‘mouth bandage’. In here, Mikulicz described a one-layered mask made of gauze [10].

Mikulicz, who had already been responsible for the introduction of sterile gloves made from cloth, noted concerning the applicability of surgical masks: ‘*…we breathed through it as easily as a lady wearing a veil in the streets…’*

Mikulicz’ assistant Hübner resumed the topic and described a two-layered mouth protection made of gauze that should prevent driblet spread. More studies regarding the germ content in the operating room air followed [11, 12].

**Fig. 2:**
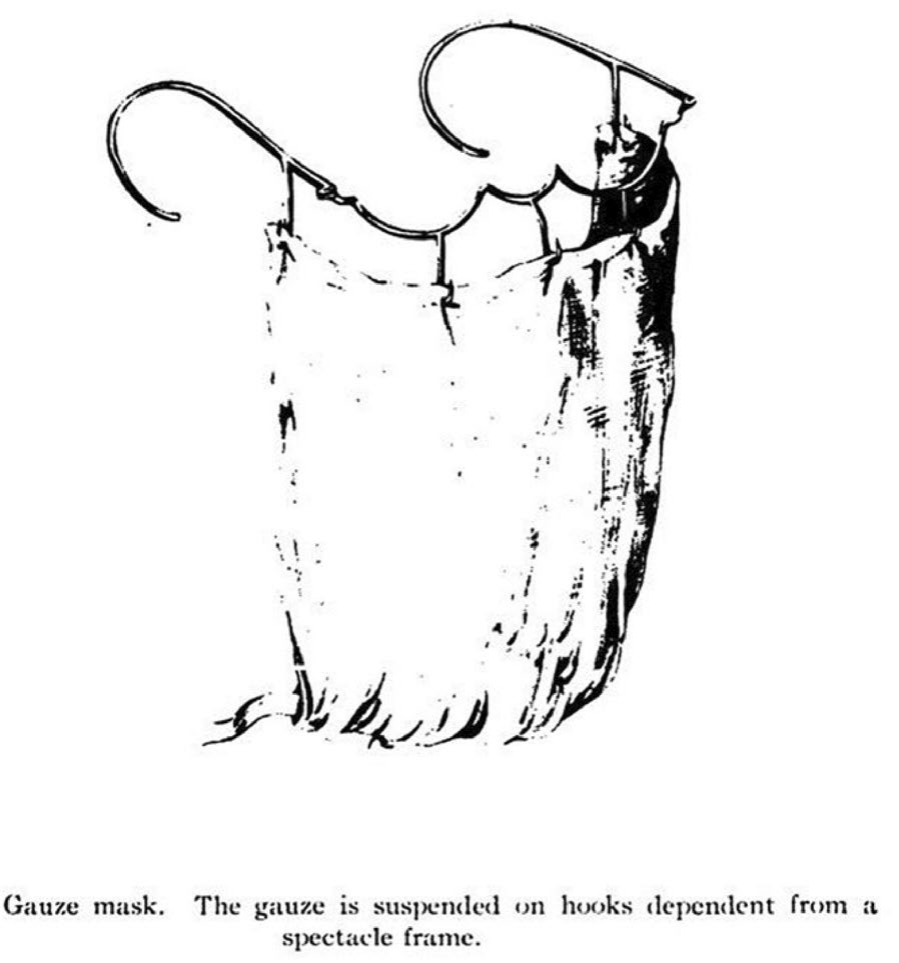
Face mask following Berkeley George Andrew Moynihan (1865–1936) Abdominal Operations 1906 Saunders, Philadelphia Vol I S 24, Repro Moll-Keyn, with permission

Until 1910, the application of face covers was not common in surgery and the general hospitals. Nevertheless, an earlier illustration of a multilayer face mask made of gauze can be found in the surgical operating teachings of the British surgeon B.G.A. Moynihan (1865–1936) (Fig. 2).

### Modern area

In 1914, the surgeon Fritz König (1866–1952) noted in a handbook on surgery for general practitioners:

*“…Due to our experience of many years we consider their (mouth masks)* - *by the way quite irritating* – *use altogether unnecessary. Only those afflicted with a catarrh or angina should wear a mouth bandage when operating that is to be sterilised in steam. Speaking should be limited and the direction of the operative field avoided…”* [13]

The surgical mask was used first in the operating rooms of Germany and the USA in the 1920s. Especially in endoscopic procedures or ‘small surgery’, the mask was renounced for a long time. There was still no hint for a facemask in the book ‘assistance for operating staff’, that was widely read in German-speaking areas in 1926, while the processing of cystoscopies for instance, also taking place in the clinical use around 1900, was described extensively on several pages [14, 15]. One year later, Martin Kirschner (1879–1942), who held the chair for surgery in Heidelberg, elaborately described the necessity of wearing a facemask in his multi-volume operational theory in the chapter ‘measures to combat infections’ [16]. In the following edition of the book ‘assistance for operating staff’ published in 1935, facemasks were then mentioned [17], which can probably be related to the increased number of studies on the reduction of germs [18, 19].

**Fig. 3:**
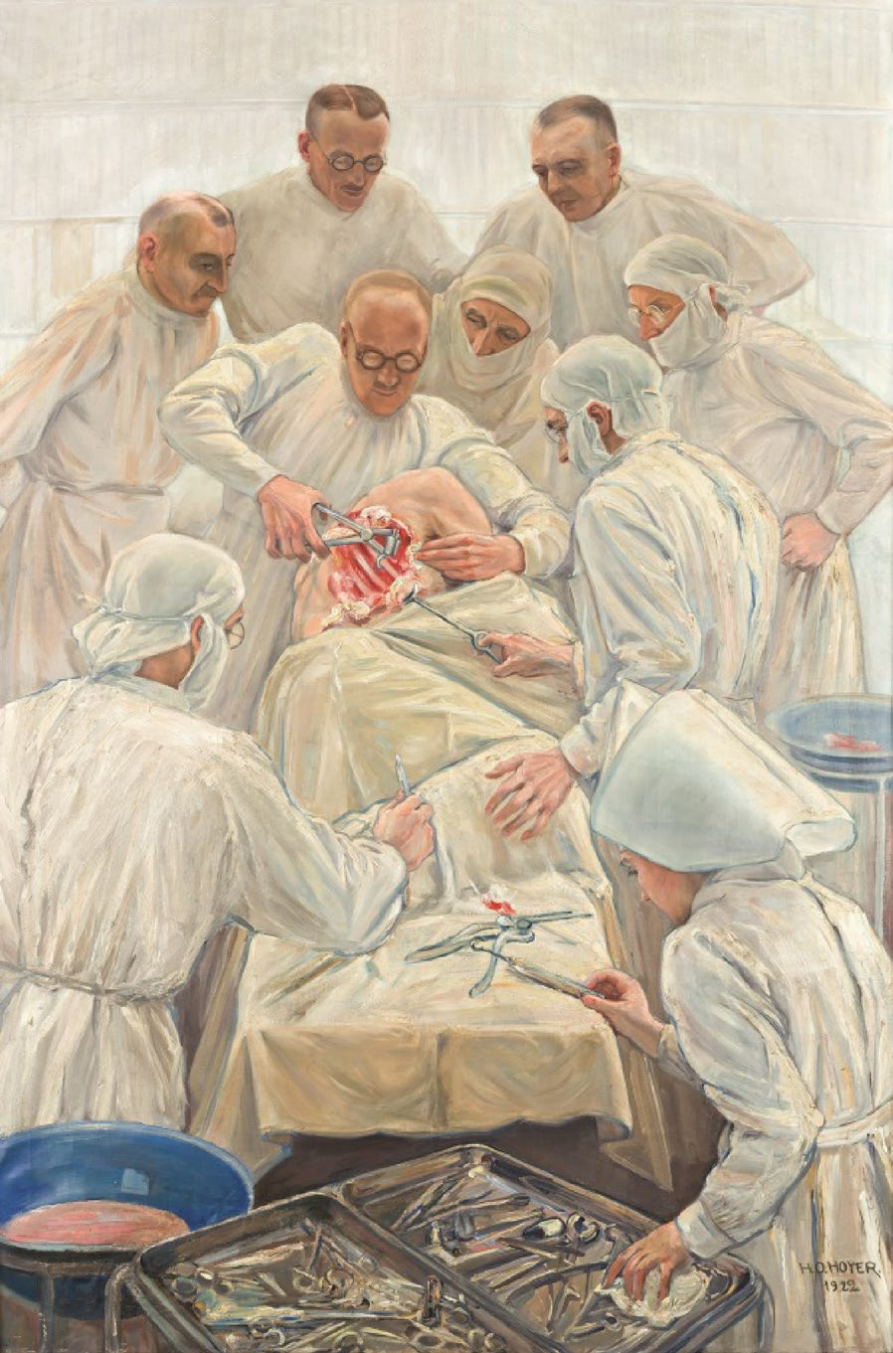
Hermann Otto Hoyer (1894–1968) 1922 Sauerbruch in a thoracotomy, Museum of Medical History at the Charité, art collection Charité, picture Bruns Inv.-Nr. 123330 Repro Moll-Keyn, with kind permission

**Fig. 4:**
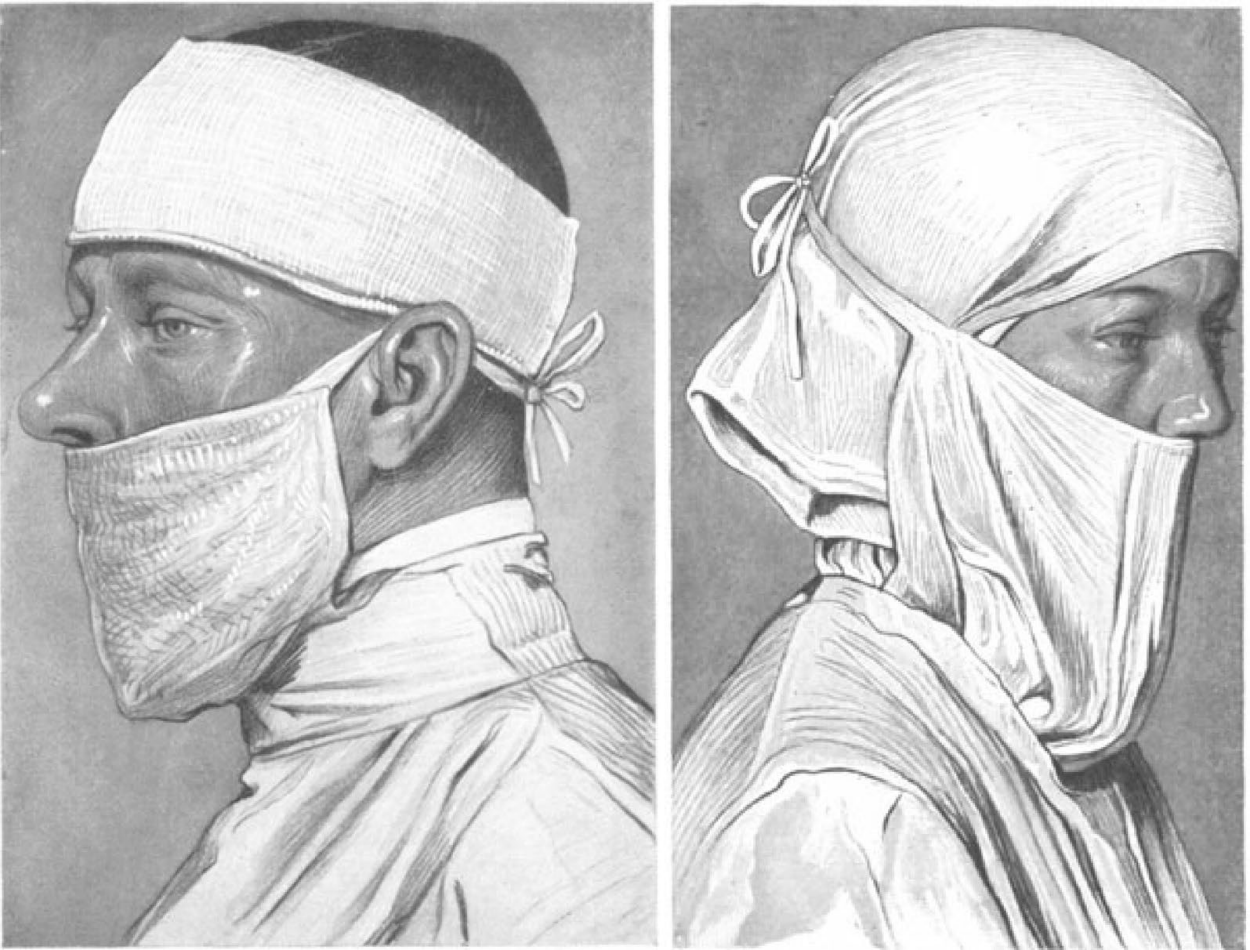
‘Our introduced face mask and forehead bandage’ and ‘face mask for person with long hair’ from: Kirschner, M. Allgemeine und Spezielle Chirurgische Operationslehre Bd 1, Julius Springer, Berlin, 1927 S. 222, Repro Moll- Keyn, with kind permission. At this time, it was not common covering the nose with the cloth-made mask

**Fig. 5:**
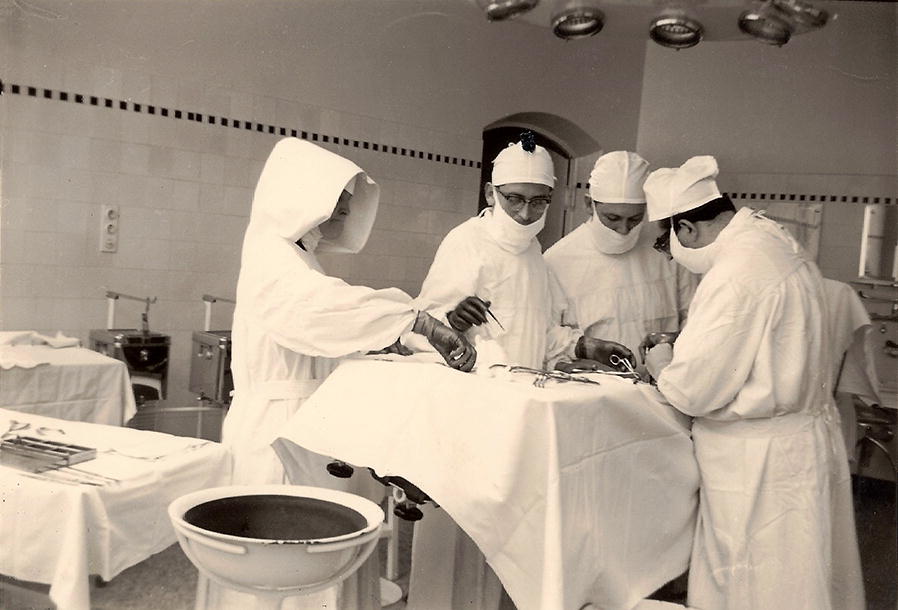
Dr. med. Ewald Matuschek and his team (source: PD Dr. med. Christiane Matuschek, daughter of Dr. med. Ewald Matuschek)

A similar situation applies for the United States. In that country, following the First World War, more and more research addressed facemasks with varying thickness [20–23]. Still, masks were not generally accepted, which can be seen in contemporary photographs [24] or paintings (Figs. 3, 4 and 5). While interns and nurses were already wearing facemasks made of cloth or gauze, the generation of head physicians rejected them, as well as rubber gloves, in all phases of an operation, as they were considered “irritating”.

In the middle of the 1930s, the research on the role of facemasks was continued in Germany and the USA [25, 26]. Only in the 1940s, washable and sterilizable masks gained acceptance in German and international surgery with only the number of gauze layers varying (2–3, 3–4) [27, 28].

Beginning in the mid-1960s, the use of disposable items made of paper and fleece was introduced all over the world after this was started in the USA.

Still in the 1990s, there were only uncertain data available. Therefore, an unresolved discussion was present between surgery and hospital hygiene, if wound infections could be reduced by the use of surgical mouth and nose protection [29, 30]. Today, following the recommendations of the RKI (German Robert Koch-Institute for hygiene), the available data indicate that surgical facemasks lower the contamination of indoor air [31].

## Conclusion

During the COVID-19 pandemic, the use of facemasks seems to be an accepted procedure worldwide although a scientific discussion is going on up to now, which has its roots in the history of medicine and science. Future research on efficiency and efficacy of long-term mask wearing outside of hospital settings is warranted and will allow for insights that are more detailed.

## Data Availability

All data and materials can be accessed via CM and FM.

## Abbreviations

COVID-19: Coronavirus Disease 2019
RKI: Robert Koch Institute
SARS-CoV-2: Severe Acute Respiratory Syndrome Coronavirus 2
USA: United States of America
